# The AusAB non-ribosomal peptide synthetase of *Staphylococcus aureus* preferentially generates phevalin in host-mimicking media

**DOI:** 10.1128/mbio.00845-24

**Published:** 2025-05-05

**Authors:** Adriana Moldovan, Markus Krischke, Claudia Huber, Clara Hans, Martin J. Müller, Wolfgang Eisenreich, Thomas Rudel, Martin J. Fraunholz

**Affiliations:** 1 Biocenter, Chair of Microbiology, University of Würzburg, Würzburg, Germany; 2 Biocenter, Chair of Pharmaceutical Biology, University of Würzburg, Würzburg, Germany; 3 Bavarian NMR Center-Structural Membrane Biochemistry, Department of Bioscience, School of Natural Sciences, Technical University of Munich, Munich, Germany; MedImmune, Gaithersburg, Maryland, USA

**Keywords:** *Staphylococcus aureus*, non-ribosomal peptide synthetases, aureusimines, phevalin, tyrvalin

## Abstract

Non-ribosomal peptide synthetases (NRPSs) are modular multidomain enzymes responsible for the biosynthesis of various secondary metabolites in an mRNA template-independent manner. They are predominantly present in bacteria and fungi, where they synthesize a variety of products, including antibiotics, siderophores, toxins, and signaling molecules. The human pathogen *Staphylococcus aureus* possesses one single NRPS, AusA, highly conserved in all sequenced *S. aureus* strains. AusA incorporates the aromatic amino acids (AAAs) phenylalanine or tyrosine, as well as the branched-chain amino acids valine and leucine into three cyclic dipeptides collectively called aureusimines: phevalin, tyrvalin, and leuvalin. By using targeted metabolomics, we found that during growth in the common tissue culture medium RPMI1640, AusA preferentially synthesizes phevalin, despite similar availability for both phenylalanine and tyrosine. Upon cultivation in a chemically defined medium, however, the yields for both products are comparable, albeit with a slight preference for phevalin. Moreover, omission of either “building block” (phenylalanine, tyrosine, or valine) does not abrogate aureusimine biosynthesis, showing that *de novo* biosynthesis of these amino acids is sufficient to yield aureusimine production. Cultivation of *S. aureus* in a synthetic medium mimicking human nasal secretions, lacking tyrosine, results in marked phevalin production, despite moderate bacterial growth. Our report on culture medium composition-driven AAA incorporation by a bacterial NRPS provides a useful basis for linking bacterial cell metabolic status to the biosynthesis of secondary metabolites.

**IMPORTANCE:**

Peptide and protein synthesis are fundamental processes in nature which are largely mediated by the ribosomal machinery. An alternative pathway for peptide synthesis is non-ribosomal mRNA template-independent synthesis, performed by so-called NRPSs. NRPSs are multi-enzyme complexes which serve the simultaneous role of template and biosynthetic machinery. They are mostly found in bacteria and fungi and are responsible for the biosynthesis of many pharmacologically significant products, including antibiotics, anticancer compounds, or immunosuppressants. The human pathogen *S. aureus* possesses one such NRPS, AusA, which synthesizes three cyclic dipeptides termed “aureusimines” using the aromatic amino acids phenylalanine and tyrosine and the branched-chain amino acids leucine and valine. Although the biological role of aureusimines remains unknown, AusA appears to play a role in the interaction of *S. aureus* with the host. In addition, owing to its minimal canonical NRPS structure and autonomous function (i.e., most NRPS pathways require the assembly of several NRPS proteins), AusA represents an excellent model system for studying such molecular assembly lines. Our observation is, to our knowledge, the first report of an NRPS showing preferential incorporation of aromatic amino acids, despite their similar availability.

## OBSERVATION

Non-ribosomal peptide synthetases (NRPSs) are large, modular multi-enzyme complexes responsible for the production of complex peptide natural products with diverse properties such as antibiotics, antifungal reagents, siderophores, pigments, or toxins (1). They are widespread across all three domains of life, but gene clusters encoding NRPSs are more commonly reported in bacteria (2). NRPSs employ a modular architecture wherein each module contains several domains responsible for introducing one amino acid into the building peptide. A canonical NRPS module contains three domains: an adenylation (A) domain, which is responsible for the selection and activation of an amino acid; a thiolation (T) domain, onto which the amino acid is covalently tethered as a thioester to the terminal thiol of 4′-phosphopantetheine prosthetic group; and a condensation (C) domain, which catalyzes peptide bond formation. Terminal modules include domains dedicated to the release of the nascent peptide either by the activity of a thioesterase domain, via cyclization, or, more rarely, by a reductase domain (R) (1, 3, 4). Non-ribosomal peptide synthesis begins with an essential post-translational modification of the NRPS itself by a phosphopantetheine transferase (PPTase), which adds a 4′-phosphopantetheine moiety to a conserved serine residue within the apo-T domains to yield functional holo-T domains (1, 5).

**Fig 1:**
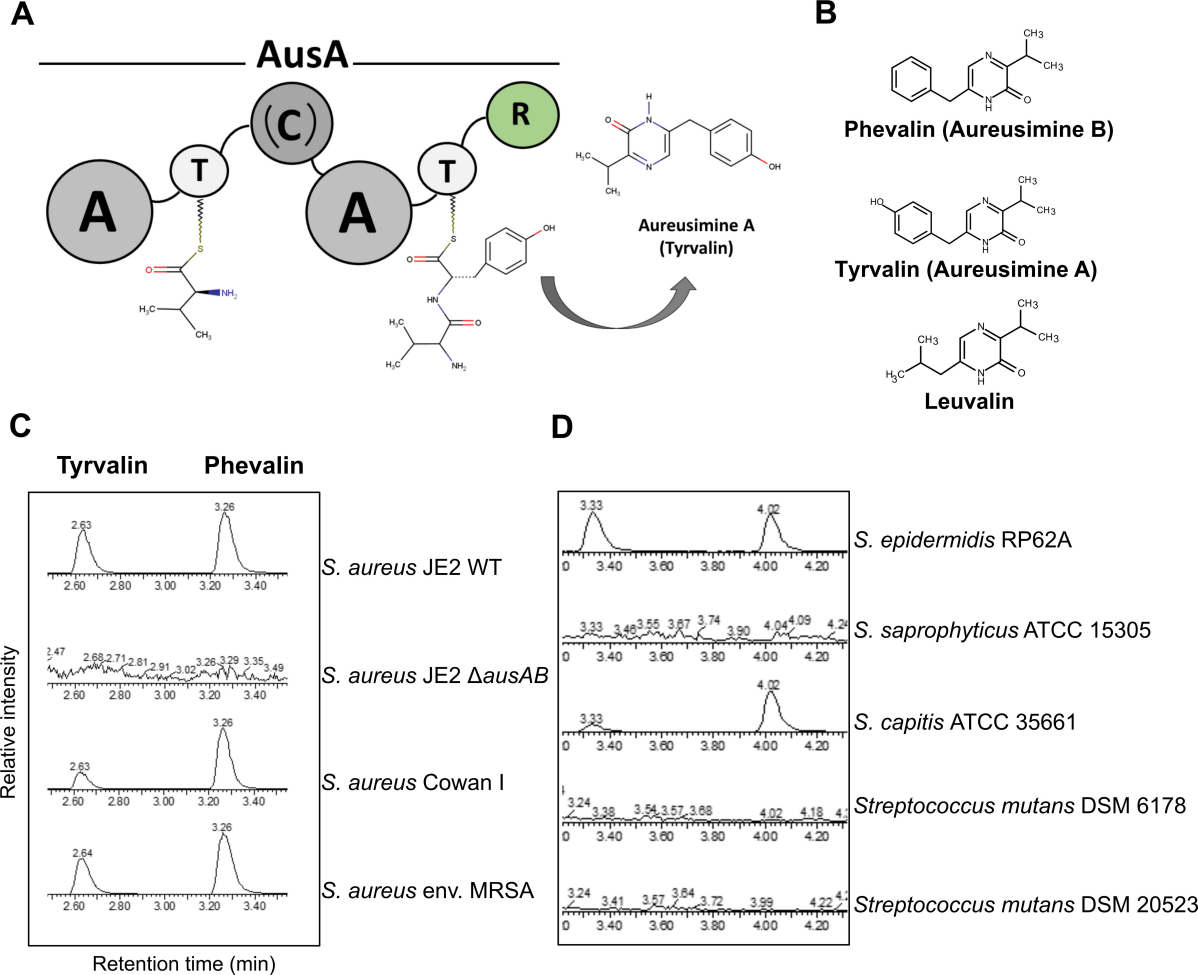
Aureusimines are synthesized by different *S. aureus* strains and by skin-associated staphylococci. (**A**) Schematic representation of the AusA non-ribosomal peptide synthetase (genomic locus ID: SAUSA300_RS00950). AusA is a ~273 kDa soluble protein with the bimodular architecture A_1_-T_1_-C-A_2_-T_2_-R. (**B**) Aureusimines (phevalin, tyrvalin, and leuvalin) are cyclic dipeptides with monoketopiperazine structure. (**C**) Phevalin and tyrvalin are synthesized by both cytotoxic (*S. aureus* JE2) and non-cytotoxic *S. aureus* (Cowan I), as well as by environmental *S. aureus*. (**D**) Phevalin and tyrvalin are detected in *Staphylococcus epidermidis* and *Staphylococcus capitis*, two skin-associated coagulase-negative staphylococcal species, but not in *Staphylococcus saprophyticus* and *Streptococcus mutans*. Phevalin and tyrvalin were detected by ultra-performance liquid chromatography–mass spectrometry (UPLC-MS) in stationary-phase culture supernatants of bacteria grown in tryptic soy broth (TSB) for 24 h. Commercially available phevalin and tyrvalin serve as standards. Leuvalin is to date not commercially available; therefore, it was not analyzed in the present study. The UPLC spectra shown are representative of three independent biological replicates (*n* = 3). (A: adenylation; T: thiolation; C: condensation; R: terminal reductase; WT: wild type; env.: environmental; MRSA: methicillin-resistant *Staphylococcus aureus*; ATCC: American Type Culture Collection).

The genome of the prominent opportunistic pathogen *Staphylococcus aureus* encodes a single NRPS biosynthetic cluster, *ausAB* (locus tags SAUSA300_RS00950 and RS00955; genome accession number NC_007793). AusA is a 277 kDa two-module, six-domain NRPS with the following domain architecture: A_1_-T_1_-C-A_2_-T_2_-R (4). apo-AusA is post-translationally modified by the AusB PPTase (Fig. 1A).

AusA is responsible for the biosynthesis of three cyclic dipeptides, formed through the condensation of the aromatic amino acids (AAAs) L-phenylalanine (Phe) or L-tyrosine (Tyr) and, to a lesser extent, the branched-chain amino acid (BCAA) L-leucine, with the BCAA L-valine (Val), to form three cyclic dipeptides with a monoketopiperazine structure: phevalin, tyrvalin, and leuvalin, collectively termed aureusimines (6, 7) (Fig. 1B).

The high degree of conservation of the *ausAB* gene cluster across all sequenced *S. aureus* strains suggests an important biological function for the NRPS. Two studies propose a role for AusAB for intracellular *S. aureus* virulence (8, 9). Furthermore, the dipeptide aldehyde form of phevalin (i.e., the non-oxidized dihydropyrazinone, released by the C-terminal reductase domain of the NRPS) has been ascribed protease-inhibitory functions, in anaerobic gut microbiota species (10). However, the exact function of any naturally occurring monoketopiperazine remains elusive.

Host niches are often scarce in amino acids (11). Here we sought to investigate the biosynthesis of aureusimines under conditions where its constituent amino acids are depleted.

The *ausAB* locus is well conserved in all sequenced *S. aureus* strains. We therefore assessed by ultra-performance liquid chromatography–mass spectrometry (UPLC-MS) several *S. aureus* strains for phevalin and tyrvalin production during growth in the complex medium tryptic soy broth (TSB). Since *ausAB* genes were shown to be upregulated during stationary phase (8), we measured aureusimine production in the stationary phase of bacterial growth. The methicillin-resistant USA300 JE2 *S. aureus* strain, the non-cytotoxic, *agr-*negative strain Cowan I (ATTC 12598), as well as an environmental *S. aureus* strain were aureusimine producers (Fig. 1C; Fig. S1).

AusA homologs of different identity levels were identified in the human-associated *Staphylococcus epidermidis*, *Staphylococcus capitis*, *Staphylococcus schweitzeri*, *Staphylococcus warneri*, and *Staphylococcus lugdunensis* (6). The degree of homology of the AusA protein was not higher than ~53% to 54%; however, aureusimine production was detected in *S. epidermidis* and *S. capitis* (Fig. 1D; Fig. S2), as well as in *S. lugdunensis* (B. Krismer, Eberhard Karls University of Tübingen, personal communication). *Staphylococcus saprophyticus*, which does not harbor *ausA* homologs (6), did not produce aureusimines. Two different *Streptococcus mutans* strains*,* a member of the human oral bacterial flora previously reported to synthesize phevalin, despite not encoding the *ausAB* cluster (12), also did not produce aureusimines under our tested conditions. Since *S. epidermidis*, *S. capitis*, and *S. lugdunensis* are human-associated staphylococci (13–15), it is tempting to speculate that aureusimine production is associated with human commensal bacteria. This is supported by a study which found phevalin, as well as several other cyclic dipeptides, to be produced by NRPSs in human gut bacteria (10).

**Fig 2:**
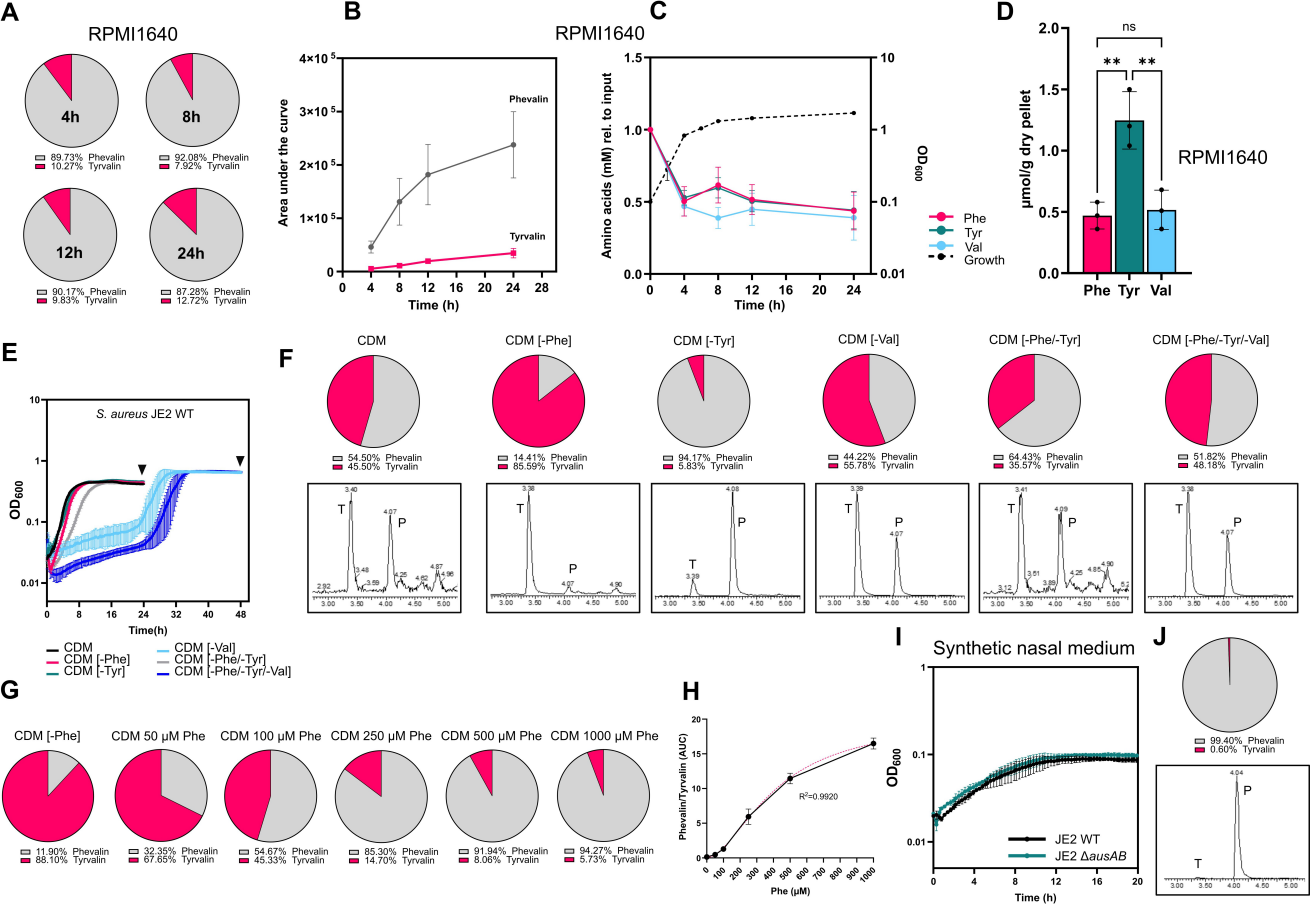
Aureusimine biosynthesis is dictated by aromatic amino acid availability in *S. aureus* (**A**) Phevalin and tyrvalin detection by UPLC-MS in *S. aureus* JE2 WT supernatants grown in commercial RPMI1640 medium for 4, 8, 12, or 24 h. (**B**) Dynamics of phevalin and tyrvalin production in commercial RPMI1640 medium. Plotted data represent raw values for area under the curve (arbitrary units) for UPLC-MS chromatograms. Shown data are mean values ± SD from independent biological replicates (*n* = 3). Same data were used to generate pie charts in panel** A**. (**C**) Depletion of Phe, Tyr, and Val during growth of *S. aureus* JE2 WT in commercial RPMI1640, normalized to input medium (left *Y* axis). *S. aureus* JE2 WT growth in RPMI1640 (right *Y* axis). OD_600_ was measured at 2, 4, 6, 8, 12, and 24 h of growth. Data are shown as mean values ± SD from independent biological replicates (*n* = 3). (**D**) Quantification of bacterial pellet-associated Phe, Tyr, and Val for *S. aureus* JE2 WT cultivated for 24 h in commercial RPMI1640 medium (micromoles amino acid per gram dry pellet). Bar graphs represent mean values ± SD from independent biological replicates (*n* = 3). Norvaline was used as the internal standard for quantification. Statistical analysis: one-way ANOVA with Tukey’s multiple comparison test (**P < 0.01; ns = not significant). (**E**) *S. aureus* JE2 WT growth in chemically defined media (CDMs), lacking Phe, Tyr, Val, Phe/Tyr, or Phe/Tyr/Val. OD_600_ was measured every 18 minutes for 24 or 48 h. Data are shown as mean values ± SD from independent biological replicates (*n* = 3). Arrows indicate the time points chosen for the aureusimine analysis shown in panel **F**. (**F**) Detection of phevalin and tyrvalin by UPLC-MS in *S. aureus* JE2 WT supernatants grown in CDM lacking Phe, Tyr, Val, and Phe/Tyr or Phe/Tyr/Val for 24 or 48 h (in case of CDM [−Val] and CDM [−Phe/−Tyr/−Val]). (**G**) Phevalin and tyrvalin detection by UPLC-MS in *S. aureus* JE2 WT supernatants grown in CDM containing different Phe concentrations. (**H**) Phe concentration-dependent changes in phevalin production (sigmoidal 4PL fit, where the *X* axis represents Phe concentrations in micromolar, and the *Y* axis represents the phevalin:tyrvalin ratio calculated from the area under the curve [AUC] for UPLC-MS chromatograms). (**I**) Growth of *S. aureus* JE2 WT and Δ*ausAB* in a synthetic nasal medium. OD_600_ was measured every 18 minutes for 20 h. Data are shown as mean values ± SD from independent biological replicates (*n* = 3). (**J**) Phevalin and tyrvalin detection by UPLC-MS in *S. aureus* JE2 WT supernatants grown in synthetic nasal medium. All pie charts represent the fitted relative area under the curve (arbitrary units) for UPLC-MS chromatograms, where tyrvalin is normalized to phevalin, which is set to 100%. Data used for pie chart generation represent mean values from independent biological replicates (panels A, F, and G, *n* = 3; panel J, *n* = 4). Representative total ion chromatograms are shown in panels F and J. (*P: phevalin; T: tyrvalin*).

Aureusimines are enigmatic natural products, and while AusAB appears to play a role in the virulence of intracellular *S. aureus* (8, 9), the exact targets of these molecules are poorly understood. Naturally occurring phevalin was first isolated from *Streptomyces* sp. and exhibited calpain inhibitory activity (16). Synthetic phevalin was shown to inhibit human neutrophil activation and enhance the phagosomal escape of *S. aureus* in epithelial cells (8), thus suggesting potential host targets for this molecule. Moreover, phevalin could be extracted from host cells infected with *S. aureus* (8), suggesting that aureusimines are likely to be present at host–pathogen interaction sites.

We sought to gain insight into the biosynthesis of aureusimines under defined conditions; therefore, we made use of the commercially available tissue culture medium RPMI1640, which contains 90 µM Phe, 110 µM Tyr, and 170 µM Val (Table S1).

We cultivated wild-type (WT) *S. aureus* JE2 in RPMI1640 for 24 h, examined tyrvalin and phevalin production over time, and observed a preference for phevalin over tyrvalin production at all analyzed time points (~87% vs ~13% phevalin-to-tyrvalin ratio), despite the slightly lower Phe concentration in the medium (Fig. 2A and B; Fig. S3 and S4). Since leuvalin is not commercially available and it represents a minor by-product, we excluded it in this study.

We next analyzed whether differences in the consumption of these amino acids could account for the differences in aureusimine production. Measurements of amino acids from supernatants at 0, 4, 8, 12, and 24 h of growth indicated a similar depletion of both Phe and Tyr as early as after 4 h of growth (Fig. 2C). Moreover, the total availability of Phe, Tyr, and Val associated with bacterial pellets after 24 h of growth indicated the presence of higher amounts of Tyr than either Phe or Val (Fig. 2D). Despite lacking the capacity for aureusimine biosynthesis, the Δ*ausAB* mutant showed comparable amino acid consumption (Fig. S5A) as well as pellet-associated levels of Phe, Tyr, and Val to the wild type (Fig. S5B).

We next sought to further understand the nutritional requirements for aureusimine biosynthesis under amino acid-depleted conditions. We therefore grew wild-type *S. aureus* JE2 in either a complete chemically defined medium (CDM; Table S1) or a CDM where Phe, Tyr or Val, or combinations thereof were excluded (Fig. 2E) and again analyzed the production of aureusimines.

Of note, bacterial growth patterns under either Phe or Tyr deprivation, as well as upon exclusion of both amino acids, resulted in a moderate delay when compared to growth in complete CDM. Consistent with previous reports (17), growth under all valine-deprived conditions was dramatically impacted, with *S. aureus* undergoing a 24 h lag phase before entering logarithmic growth. We observed a similar growth pattern when all three amino acids, Phe, Tyr, and Val, were excluded in media of both wild-type JE2 (Fig. 2E) and its ∆*ausAB* mutant (Fig. S5C).

Upon cultivation in complete CDM containing 100 µM of Phe, Tyr, and Val (each), tyrvalin and phevalin were detected in comparable amounts in stationary-phase cultures, albeit with a slight phevalin preference (Fig. 2F; Fig. S6A).

Upon exclusion of Phe from the medium (CDM [−Phe]), we observed enhanced tyrvalin production but a stark reduction in phevalin production (Fig. 2F; Fig. S6B). Conversely, upon exclusion of Tyr from the CDM formulation (CDM [−Tyr]), phevalin was detected as a major product, while tyrvalin was only detected in trace amounts in bacterial supernatants (Fig. 2F; Fig. S6C). Cultivation under Val depletion (CDM [−Val]) resulted in the production of both aureusimines in comparable amounts (Fig. 2F; Fig. S6D).

We observed that the exclusion of all three “building blocks” for aureusimine production (Phe, Tyr, and Val) from the CDM formulation resulted in similar phevalin-to-tyrvalin ratios as during growth in complete CDM (Fig. 2F; Fig. S6F), with a slight preference for phevalin over tyrvalin. However, if both Phe and Tyr, but not Val, were excluded from CDM, phevalin was, again, the favored product (~64% vs ~36% phevalin-to-tyrvalin ratio) (Fig. 2F; Fig. S6E).

Importantly, whenever grown under either Phe, Tyr, or Val-depleted conditions, *S. aureus* could synthesize the respective amino acid (Fig. S7), suggesting that *de novo* amino acid biosynthesis is sufficient to fuel aureusimine production.

Upon comparison of aureusimine production during growth in RPMI1640 vs CDM, we noticed a discrepancy regarding the phevalin-to-tyrvalin ratios that we measured under the different conditions. Despite both media containing comparable amounts of Phe and Tyr (90 µM Phe and 117 µM Tyr in RPMI1640, vs 100 µM of both Phe and Tyr in CDM), phevalin appeared to be the preferred product (~87% phevalin) in RPMI1640, whereas in CDM, both products were present in comparable amounts (~54% phevalin and 45% tyrvalin). An increase in exogenous Phe provided in the CDM led to an incrementally higher production of phevalin compared to tyrvalin (Fig. 2G and H; Fig. S8), indicating that amino acid availability impacts the production of aureusimines.

Since amino acid concentrations appeared to be decisive for the type of aureusimine that will be produced, we wondered whether aureusimines would also be synthesized in host niches known to be nutrient deplete. Human nasal secretions were shown to completely lack several amino acids, including methionine, glutamine, isoleucine, asparagine, and aspartate, along with the tyrvalin component, tyrosine (11). We therefore used an established synthetic nasal medium (SNM) (11) and checked for aureusimine production. We found that, despite the severe growth defect in both strains JE2 WT and ∆*ausAB* (Fig. 2I), phevalin was readily produced by the wild type, whereas tyrosine was only detectable in trace amounts (Fig. 2J; Fig. S9).

We conclude that, dependent on the host/environmental niche where *S. aureus* is found, the AusA NRPS might favor phevalin vs tyrvalin biosynthesis. Preference for phevalin over tyrvalin, when both Phe and Tyr are present in equal amounts, which is more apparent during growth in RPMI1640 than in CDM, suggests that the general metabolic state of the bacterium might regulate AusA activity and/or aureusimine production.

Within the AusA NRPS, the A_1_ domain activates and loads T_1_ with L-Val to yield L-Val-S~T_1_; the A_2_ domain then activates and loads T_2_ with L-tyrosine to yield L-Tyr-S~T_2_. The C domain subsequently catalyzes peptide bond formation, which results in the dipeptide L-Val-L-Tyr~T_2_, which is then reduced by the R domain to an intermediate amino aldehyde that cyclizes to an imine. Oxidation to the pyrazinone final product likely occurs spontaneously (4).

The BCAA Val is present in all three synthesized aureusimines and is the only natural substrate for the A_1_ domain (4). BCAAs (isoleucine, leucine, and valine) are essential metabolites and “expensive” goods for *S. aureus*. BCAAs are incorporated into membrane branched-chain fatty acids (18), where they comprise 65% of membrane fatty acids (19), but also represent the most abundant amino acids in staphylococcal proteins (20). In *S. aureus*, BCAA acquisition and biosynthesis are stringently monitored by the global regulator CodY (21), which uses isoleucine and GTP as co-factors (17, 22). Transcription of genes involved in BCAA import (*brnQ1*, *brnQ2*, and *bcaP*) and biosynthesis (*leu* and *ilv* operons) is sequentially de-repressed upon drops in intracellular BCAAs (23, 24), with the bacterium prioritizing BCAA scavenging over *de novo* synthesis (17, 23). During growth under Val-deplete conditions, *S. aureus* responds dramatically by a prolonged lag phase (Fig. 2E). Moreover, in the absence of exogenous Val, suppressor mutants in either *codY* or the 5′ UTR of *ilvD* (first gene in the *ilv-leu* operon) are selected (17), highlighting the importance of Val availability for *S. aureus* growth.

The AAAs Phe, Tyr, and tryptophan are synthesized from the central metabolite D-erythrose 4-phosphate, via the shikimate pathway (25). Both Phe and Tyr, similarly to BCAAs, are likely used for protein synthesis but not for catabolic purposes (26). The genes encoding the enzymes and proteins for the synthesis of AAAs (*trp*, *aro*, and *tyr*) are controlled by repression via CodY; however, they are not as stringently repressed by CodY as genes involved in BCAA biosynthesis (23). The delayed growth of *S. aureus* JE2 under deprivation of either AAAs or BCAAs (Fig. 2E) highlights the importance of maintaining a stable intracellular pool of these metabolites.

It is therefore remarkable that *S. aureus* uses Val, as well as Phe and Tyr, as the building blocks for secondary metabolites which are secreted at micromolar concentration.

While the NRPS A_1_ domain has a strict specificity for L-valine, A_2_ has a relaxed substrate specificity for L-Tyr, L-Phe, or, to a lesser extent, L-leucine. The A_2_ domain of the AusA NRPS has a lower affinity to L-Phe (*K*_*M*_ = ~2 mM), compared to L-Tyr (*K*_*M*_ = ~1 mM), making L-Tyr the preferred substrate (4). Therefore, the reason for a seeming preference for phevalin over tyrvalin production during growth in RPMI1640, with a slight similar tendency during growth in CDM, remains elusive.

AAAs are commonly known to be incorporated into secondary metabolites; however, a selective preference for one over the other has not yet been reported.

### Conclusion

Here, we utilize a metabolomics approach to show that the AusA NRPS appears to preferentially fuel Phe over Tyr into cyclic dipeptides, when both amino acids are available in similar concentrations. During growth in a synthetic nasal medium, *S. aureus* produces phevalin but only trace amounts of tyrvalin.

Our observation suggests an unusual way of amino acid incorporation into secondary metabolites. The observation that modulation of amino acid concentrations is linked to biosynthetic output, opens new prospects for further explorations on NRPSs, both in *S. aureus* and other bacterial species. Moreover, the fact that *S. aureus* sequesters these amino acids by using them for aureusimine production, even under conditions of severe nutrient depletion and growth impairment (i.e., in SNM), supports an important role for these natural products in the *S. aureus*–host interaction.

## SUPPLEMENTAL MATERIAL

Supplemental Material mbio.00845-24-s0001.pdf Supplemental figures, tables, and methods.

ASM does not own the copyrights to Supplemental Material that may be linked to, or accessed through, an article. The authors have granted ASM a non-exclusive, world-wide license to publish the Supplemental Material files. Please contact the corresponding author directly for reuse.
