# Supplementary material for: The AusAB non-ribosomal peptide synthetase of Staphylococcus aureus preferentially generates phevalin in host-mimicking media
Source: mBio. 2025 May 5;16(6):e00845-24. doi: 10.1128/mbio.00845-24 (PMC12153313; doi:10.1128/mbio.00845-24)
Supplement: Supplemental Material — Supplemental figures, tables, and methods. [file mbio.00845-24-s0001.pdf]

Supplemental Materials

SUPPLEMENTAL FIGURES

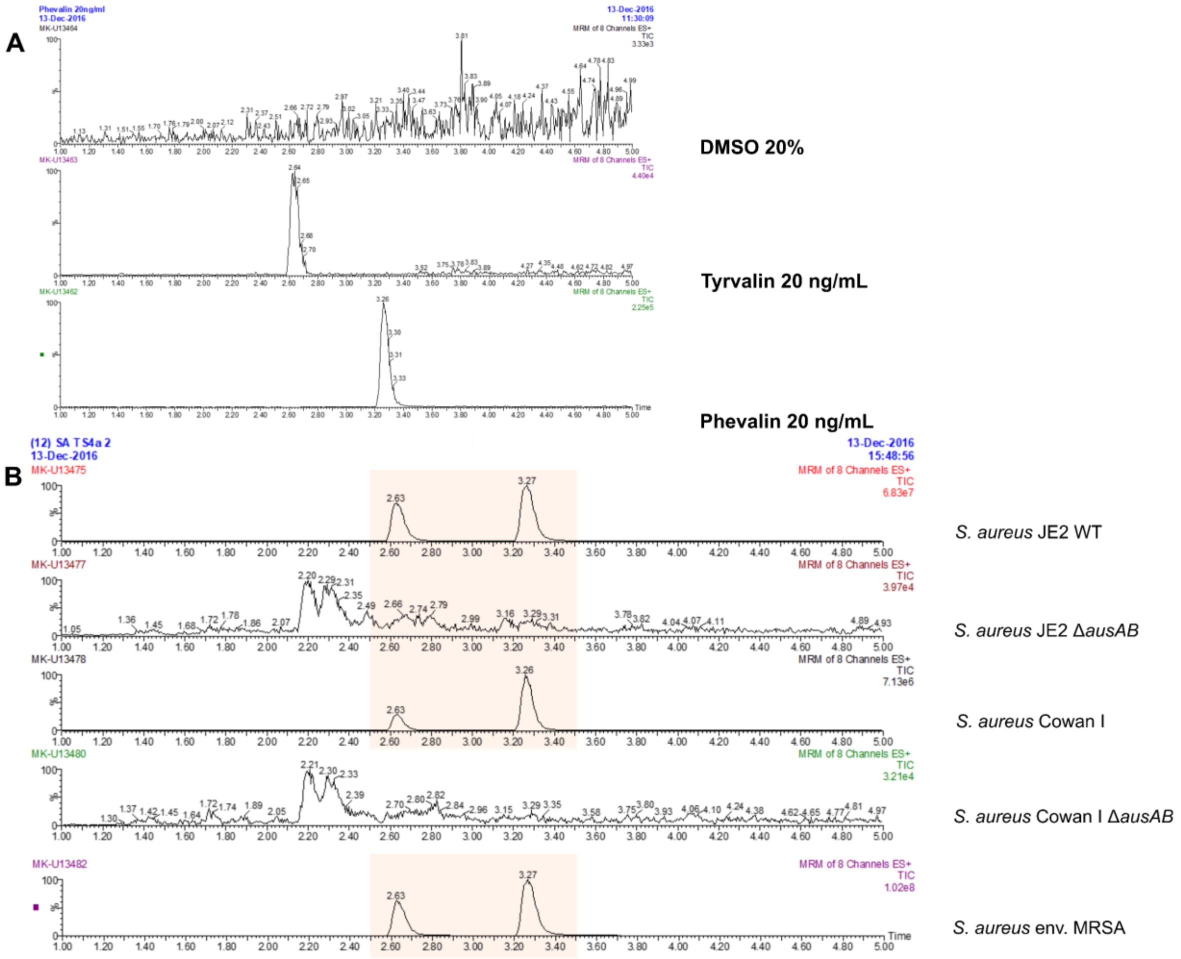

Fig. S1 UPLC chromatograms (un-cropped)

Representative UPLC chromatograms for data depicted in Fig 1C (n=3). Phevalin and tyrvalin were detected by multiple reaction monitoring (MRM). Chromatograms depict intensities per cent (Y-axis) and retention time (X-Axis, min). (**A**) Commercially available phevalin and tyrvalin were used as standards. DMSO 20% (v/v) serves as solvent control. (**B**) Box indicates cropped areas shown in Fig 1C.

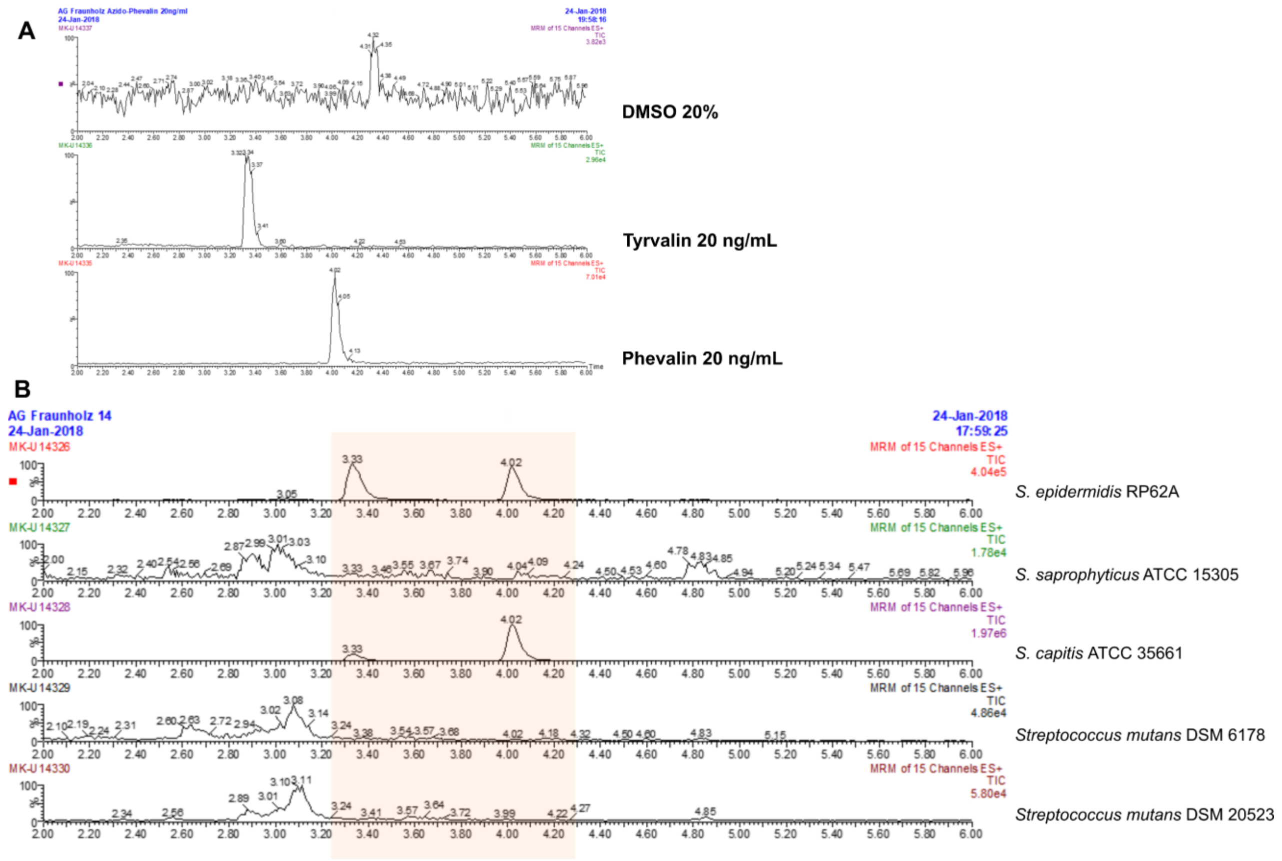

Fig. S2 UPLC chromatograms (un-cropped)

Representative UPLC chromatograms for data depicted in Fig 1D (n=3). Phevalin and tyrvalin were detected by multiple reaction monitoring (MRM). Chromatograms depict intensities per cent (Y-axis) and retention time (X-Axis, min). (**A**) Commercially available phevalin and tyrvalin were used as standards. DMSO 20% (v/v) serves as vehicle control (**B**) Box indicates cropped areas shown in Fig 1D.

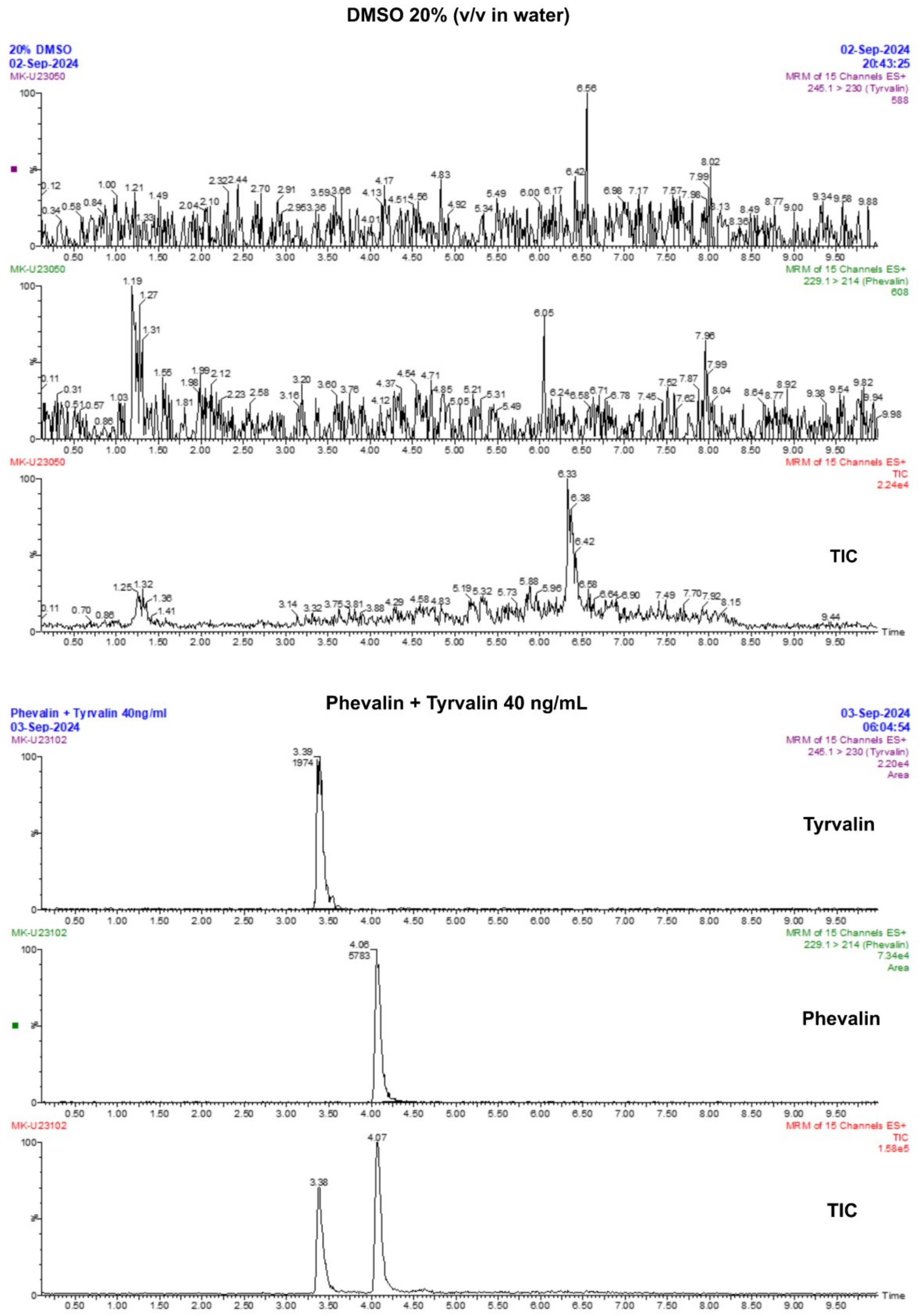

Fig. S3 UPLC chromatograms for phevalin/tyrvalin standards

Phevalin and tyrvalin were detected by multiple reaction monitoring (MRM). Chromatograms depict intensities per cent (Y-axis) and retention time (X-Axis, min). Commercially available phevalin and tyrvalin were used as standards. DMSO 20% (v/v) serves as vehicle control (*TIC*= *total ion chromatogram*).

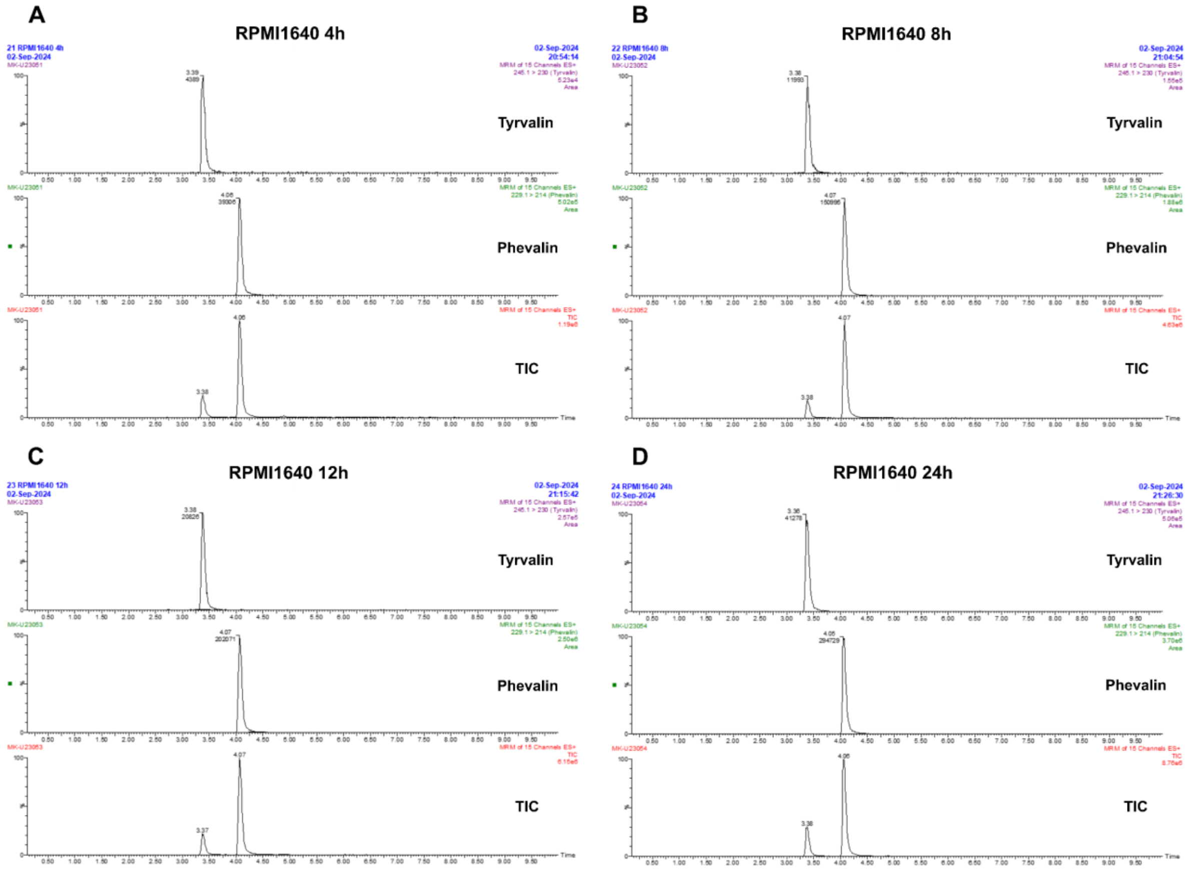

Fig. S4 Representative UPLC chromatograms for Fig 2A and B

Representative UPLC chromatograms for data depicted in Fig 2A and B (n=3). Phevalin and tyrvalin were detected by multiple reaction monitoring (MRM). Chromatograms depict intensities per cent (Y-axis) and retention time (X-Axis, min). Commercially available phevalin and tyrvalin were used as standards. DMSO 20% (v/v) serves as vehicle control (*TIC= total ion chromatogram*).

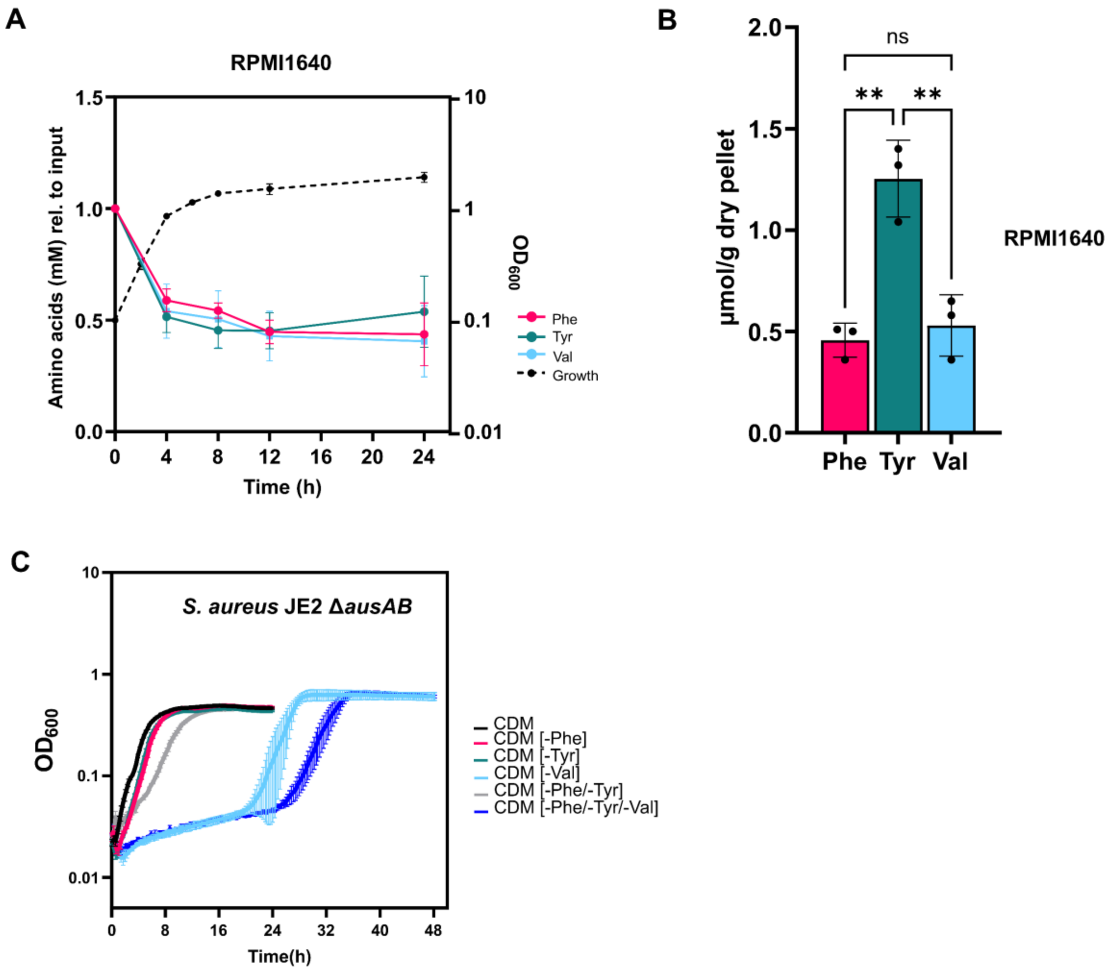

Fig. S5 Amino acid availability and growth dynamics of *S. aureus* JE2 Δ*ausAB*

(**A**) Depletion of Phe, Tyr and Val during growth of *S. aureus* JE2 Δ*ausAB* in commercial RPMI1640, normalized to input medium (left Y axis). *S. aureus* JE2 Δ*ausAB* growth in RPMI1640. OD_{600} was measured at 2, 4, 6, 8, 12 and 24h of growth (right Y axis). Data are shown as mean values ±SD from independent biological replicates (n=3). (**B**) Quantification of bacterial pellet-associated Phe, Tyr and Val for *S. aureus* JE2 Δ*ausAB* cultivated for 24h in commercial RPMI1640 medium (µmol amino acid/g dry pellet). Bar graphs represent mean values ±SD from independent biological replicates (n=3). Norvaline was used as internal standard for quantification. Statistical analysis: One-way ANOVA with Tukey's multiple comparisons test (*** p<0.01; ns=not significant*). (**C**) *S. aureus* JE2 Δ*ausAB* growth in chemically defined media (CDM), lacking Phe, Tyr, Val, Phe/Tyr or Phe/Tyr/Val. OD_{600} was measured every 18 minutes for 24 hours or 48 hours. Data are shown as mean values ±SD from independent biological replicates (n=3).

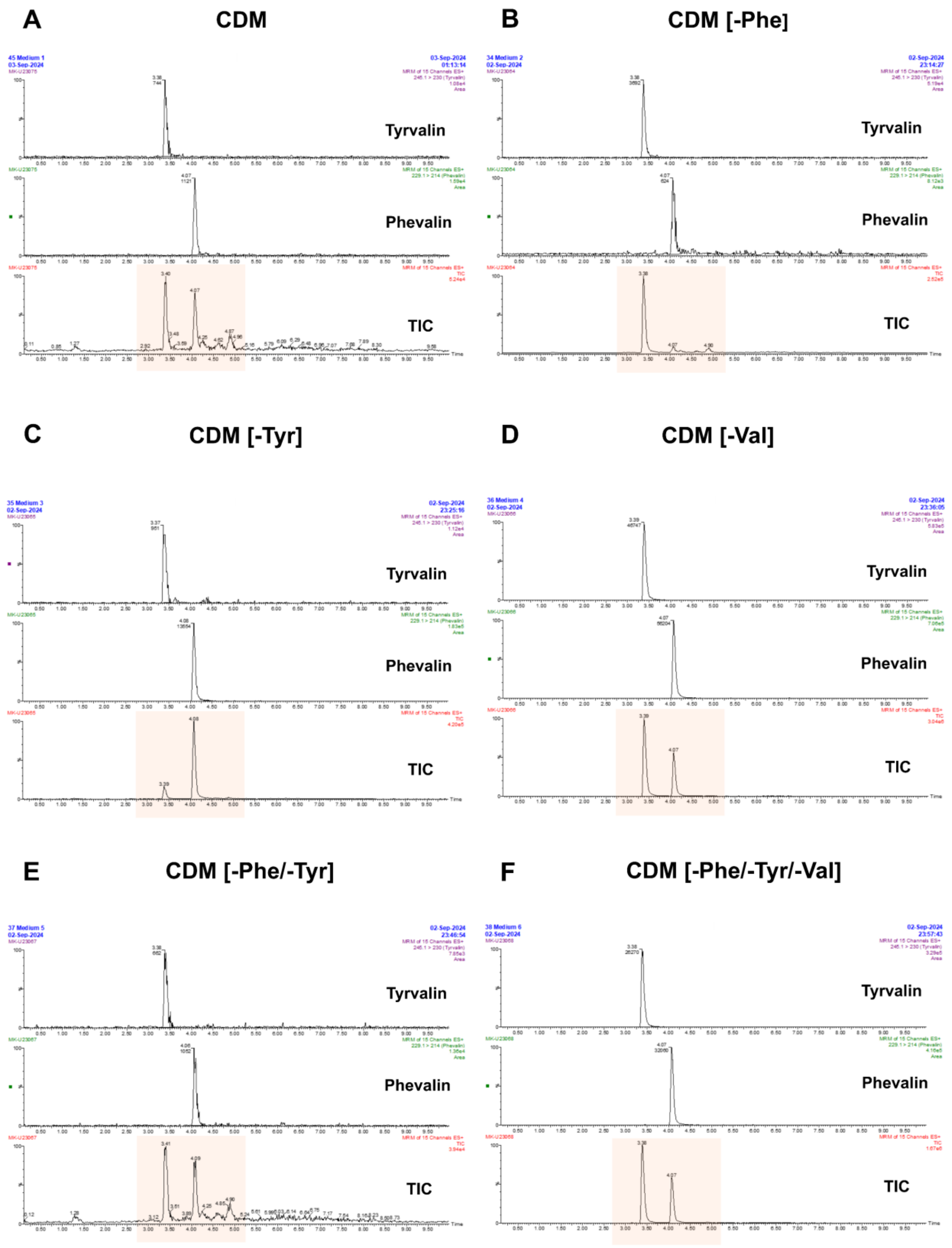

Fig. S6 Representative UPLC chromatograms (un-cropped)

Representative UPLC chromatograms for data depicted in Fig 2F (n=3). Phevalin and tyrvalin were detected by multiple reaction monitoring (MRM). Chromatograms depict intensities per cent (Y-axis) and retention time (X-Axis, min). Commercially available phevalin and tyrvalin were used as standards. DMSO 20% (v/v) serves as vehicle control (*TIC= total ion chromatogram*). Box indicates cropped areas shown in Fig 2F.

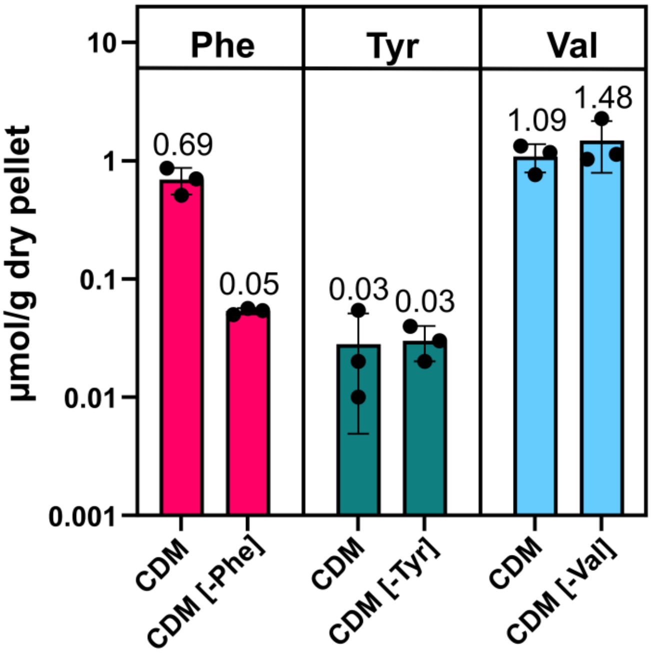

Fig. S7 ***De novo*** **amino acid biosynthesis by** ***S. aureus*** **JE2 WT**

Quantification of bacterial pellet-associated Phe, Tyr and Val for *S. aureus* JE2 WT cultivated for 24h in complete CDM, CDM [-Phe] or CDM [-Tyr] and 48h in CDM [-Val] (data are expressed as µmol amino acid/g dry pellet). Bar graphs represent mean values ±SD from independent biological replicates (n=3). Norvaline was used as internal standard for quantification.

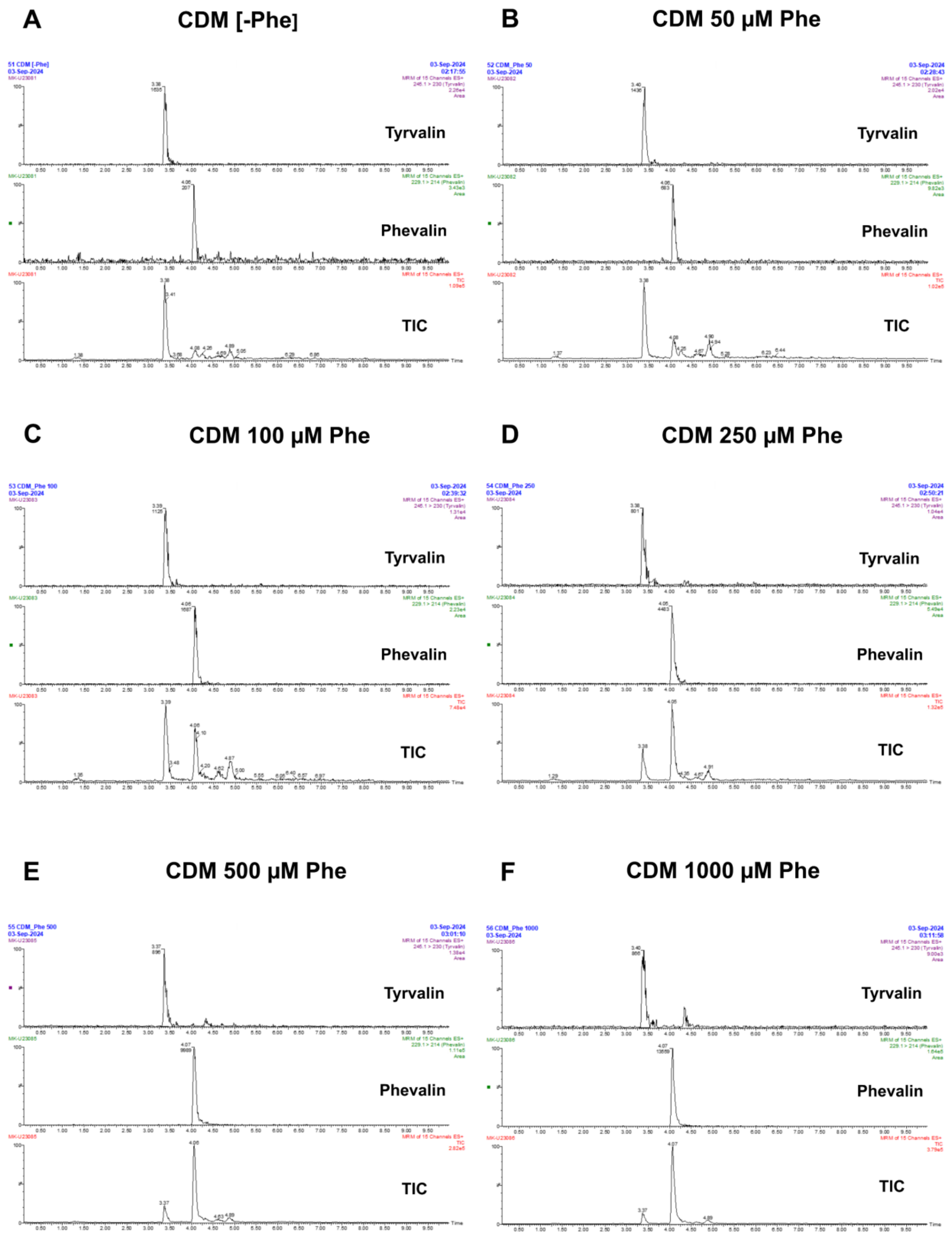

Fig. S8 Representative UPLC chromatograms (un-cropped)

Representative UPLC chromatograms for data depicted in Fig 2G (n=3). Phevalin and tyrvalin were detected by multiple reaction monitoring (MRM). Chromatograms depict intensities per cent (Y-axis) and retention time (X-Axis, min). Commercially available phevalin and tyrvalin were used as standards. DMSO 20% (v/v) serves as vehicle control (*TIC= total ion chromatogram*).

Synthetic nasal medium

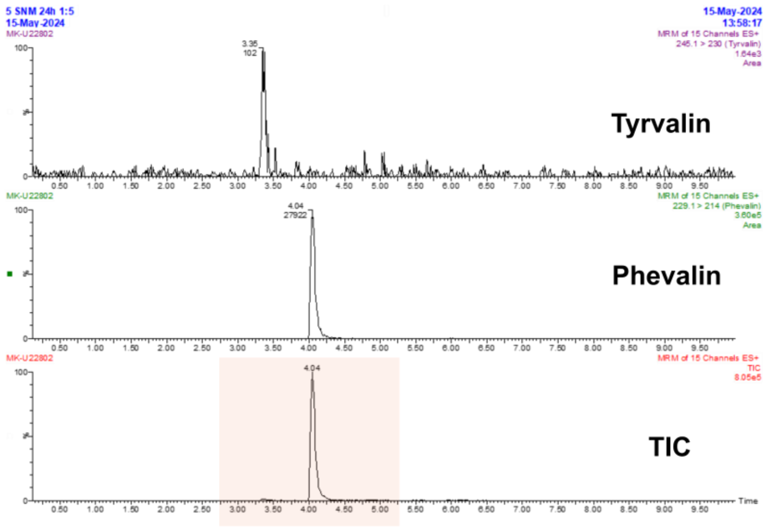

Fig. S9 Representative UPLC chromatograms (un-cropped)

Representative UPLC chromatograms for data depicted in Fig 2J (n=4). Phevalin and tyrvalin were detected by multiple reaction monitoring (MRM). Chromatograms depict intensities per cent (Y-axis) and retention time (X-Axis, min). Commercially available phevalin and tyrvalin were used as standards. DMSO 20% (v/v) serves as vehicle control (*TIC= total ion chromatogram*). Box indicates cropped area shown in in Fig 2J.

SUPPLEMENTAL TABLES

Table S1 – Cultivation media used in this study

Cells highlighted in red indicate modifications to the original medium. Cells highlighted in grey indicate a missing component in the formulation.

	RPMI 1640	CDM	SNM
	Thermo Fisher, Cat. No. 72400054	Modified from [1]	[2]
**Amino Acids (mM)**			
Glycine	0.13	1	0.150
L-Alanyl		1	0.150
L-Alanyl-Glutamine	2.06		
L-Arginine	1.15	1	0.100
L-Asparagine	0.38	1	
L-Aspartic acid	0.15	1	
L-Cystine	0.21		
L-Cysteine		1	0.010
L-Glutamic Acid	0.14	1	0.100
L-Histidine	0.10	1	0.050
L-Hydroxyproline	0.15	1	
L-Isoleucine	0.38	1	
L-Leucine	0.38	1	0.300
L-Lysine hydrochloride	0.27	1	0.150
L-Methionine	0.10	1	
L-Phenylalanine	0.09	0.100	0.150
L-Proline	0.17	1	0.150
Ornithine-HCl			0.100
L-Serine	0.29	1	0.120
L-Threonine	0.17	1	0.200
L-Tryptophan	0.02	1	0.020
L-Tyrosine	0.11	0.100	
L-Valine	0.17	0.100	0.100
**Vitamins (µM)**			
Biotin (Vit. B7)	0.82	0.120	0.082
Choline chloride	21.43		
Cyanocobalamine (Vit. B12)	0.004	0.108	0.074
D-Calcium pantothenate (Vit. B5)	0.52	0.630	0.210
Folic Acid	2.27		
i-Inositol	194.44		
Niacinamide/Nicotinamide (Vit. B3)	8.20	2.430	
Nicotinic acid (Vit. B3 vitamer)			1.62
Para-Aminobenzoic acid	7.30	0.87	0.58
Pyridoxamine hydrochloride (Vit. B6)			1.47
Pyridoxine hydrochloride (Vit. B6)	4.85	1.86	
Riboflavin (Vit. B2)	0.53	0.60	0.53
Thiamine hydrochloride (Vit. B1)	2.97	0.87	0.59
**Inorganic Salts (mM)**			
Ammonium chloride (NH4Cl)		9.25	

Calcium nitrate (Ca(NO3)2 x 4H2O)	0.42		
Magnesium sulfate (MgSO4 x H2O)	0.41	1.65	0.5
Monophosphate potassium (KH2PO4)		10	5
Potassium chloride (KCl)	5.33		20
Potassium phosphate (K2HPO4)			5
Sodium bicarbonate (NaHCO3)	23.81		
Sodium chloride (NaCl)	94.83	8.5	125
Sodium citrate tribasic dihydrate		0.14	
Sodium dihydrogen phosphate (NaH2PO4)			5
Sodium phosphate dibasic (Na2HPO4)	5.63	12.5	5
Urea			5
**Trace Elements (µM)**			
Boric acid (H3BO3)		46.25	0.10
Cobalt (II) chloride x 6 H2O			0.80
Cobalt (II) nitrate x 6 H2O		0.16	
Copper (II) chloride x 2 H2O			0.01
Copper (II) sulfate		0.32	
Iron (III) chloride (FeCl3)		0.75	
Manganese (II) chloride x 4 H2O		9.14	0.51
Nickel chloride x 6 H2O		0.1	0.10
Sodium molibdate x 2 H2O		1.83	0.15
Zinc chloride (ZnCl2)			0.51
Zinc sulphate (ZnSO4)		0.77	
**Organic acids (µM)**			
Citrate (Sodium-citrate dihydrate)			20
Fumarate (Sodium-fumarate dibasic)			5
Malate (Malic acid)			10
Pyruvate (Sodium-pyruvate)			100
Succinate (Succinic acid disodium salt x 6 H2O)			200
**Other Components**			
D-Glucose (mM)	11.11	25	2
2, 2'-Bipyridine (µM)			200
Glutathione (reduced) (mM)	0.003		
HEPES (mM)	25.03		
Phenol Red (mM)	0.01		

Table S2 - Bacterial strains used in this study

Strain	Description	Source/Reference
Staphylococcus aureus		
JE2 WT	Plasmid-cured, USA300 LAC derivative, methicillin-resistant (MRSA)	Lab stock [3]
JE2 Δ*ausAB*	*ausAB* knock out, marker-less	This study
Cowan I	NCTC 8530, isolated from septic arthritis, *agr* dysfunction, low expression of toxins and proteases	ATCC 12598, Lab stock
RN4220	Restriction-deficient derivative of NCTC 8325-4 (cured of prophages Φ11, Φ12, Φ13), β-toxin producer, no production of α-toxin or δ-toxin, phenotypically *agr*-negative	Lab stock [4]
S. aureus environmental isolate (MRSA)	Source of isolation: pig farm, Germany	[5]
Escherichia coli		
DH5α	*fhu*A2 *lac*Δ*U169 pho*A *glnV44 Φ80' lacZ*ΔM15 *gyr*A96 *rec*A1 *rel*A1 *end*A1 *thi*-1 *hsd*R17	BRL Life Technology, Lab stock
Other		
Staphylococcus epidermidis RP62A	Source of isolation: catheter sepsis	Lab stock
Staphylococcus saprophyticus ATCC 15305	Source of isolation: urine	Lab stock
Staphylococcus capitis ATCC 35661	Source of isolation: unknown	Lab stock
Streptococcus mutans DSM 6178 (ATCC 35668)	Source of isolation: unknown	DSMZ-German Collection of Microorganisms and Cell Cultures GmbH
Streptococcus mutans DSM 20523 (ATCC 25175)	Source of isolation: carious dentine	DSMZ-German Collection of Microorganisms and Cell Cultures GmbH

Table S3 - Plasmids used in this study

Plasmid	Description	Reference
pKOR1_*ausAB*	Knock-out plasmid for marker-less allelic replacement of the *ausAB* locus (SAUSA300_RS00950 – RS00955); ChlR	This study

Table S4 - Oligonucleotides used in this study

Primer name	Sequence 5'-3'	Purpose
MF663	ATTGGATGACAAACTTATCGGTAGC	Confirm *ausAB* knock-out
MF629	GATACTGTCAGACGTCATATTAACTACTC	Confirm *ausAB* knock-out
pkor_0181_for_1	GGGGACAAGTTTGTACAAAAAAGCAGGCTCCGTAATTATATGTTATTGATTTCG	pKOR1_*ausAB* deletion
pkor_0181_for_2	CCGCGGAAAAAGTCCTTCTTTCATATCATAATAC	pKOR1_*ausAB* deletion
pkor_0181_rev_1	CCGCGGGTAGGGAAAGTTATGACAGTATTTGTAATG	pKOR1_*ausAB* deletion
pkor_0181_rev_2	GGGGACCACTTTGTACAAGAAAGCTGGGTGAATTAGTGCGACAACATTTTGTCTATC	pKOR1_*ausAB* deletion
pkor_0182_for_1	GGGGACAAGTTTGTACAAAAAAGCAGGCTGGTGCTTATCTGATTGAAGTACTAC	pKOR1_*ausAB* deletion
pkor_0182_for_2	CCGCGGAACTTTCCCTACTTACTTATTGAATATTG	pKOR1_*ausAB* deletion
pkor_0182_rev_1	CCGCGGTATGACGTCTGACAGTATCATTGC	pKOR1_*ausAB* deletion
pkor_0181_rev2_1	GGGGACCACTTTGTACAAGAAAGCTGGGTCTAAAACATTTATTTACCGTTCATCTC	pKOR1_*ausAB* deletion

MATERIAL AND METHODS

Bacterial culture conditions

Bacterial strains are listed in Table S2. All liquid cultures were grown aerobically at 37°C with 200 rpm shaking. *E. coli* was grown in Luria-Bertani broth (LB) or on LB agar containing 100 µg/mL ampicillin for plasmid maintenance when necessary. *S. aureus* strains were routinely grown in tryptic soy broth (TSB), or on TSB agar plates (TSA) (Sigma Aldrich, Cat. No. T8907) if not stated otherwise. Following culture media were used: commercial tissue culture medium RPMI1640 (Thermo Fisher, Cat. No. 72400054), different formulations of a chemically defined media (CDM) based on [1] and a synthetic nasal medium (SNM) [2] (Table S1).

Generation of *S. aureus* JE2 Δ*ausAB* mutant

Marker-less deletion of the *ausAB* operon in *S. aureus* JE2 (genomic locus ID: SAUSA300_RS00950 and RS00955) was performed by allelic replacement using pKOR1 [6]. For the generation of the knock-out construct pKOR1_*ausAB*, approximately 1 kb regions upstream and downstream of the targeted loci were amplified by PCR using the "pkor" primers listed in Table S4, to generate PCR products with SacII restriction sites, as well as *att*B recombination sites. The pKOR1_*ausAB* construct was obtained by Gateway Cloning and subsequently transformed into *S. aureus* JE2 by phage transduction, via *S. aureus* RN4220. The allelic replacement protocol was carried out as described [6]. Successful seamless deletion of the *ausAB* locus was confirmed by PCR using MF663 and MF629 primers, followed by full-genome sequencing to exclude secondary-site mutations.

Bacterial growth curves

Bacterial growth curves were recorded for 12 or 48 hours in 48-well plates using a TECAN MPlex plate reader. *S. aureus* strains were grown overnight in TSB and washed 4 x in Dulbecco's Balanced Salt Solution (DPBS; Thermo Fisher, Cat. No. 14190169) before inoculation at OD_{600}=0.1, in the appropriate medium (RPMI1640, CDM or SNM). On the next day, cultures were diluted to an OD_{600}=0.1 in fresh corresponding medium and used to inoculate 400 µL/well. Sterile medium was used as reference. Absorbance at 600 nm (OD_{600}) was measured every 18 min, under continuous shaking at 200 rpm, until stationary growth phase was reached.

Aureusimine purification by chloroform extraction and UPLC-MS analysis

Aureusimines from bacterial supernatants were purified by chloroform extraction. Bacteria were grown overnight in RPMI1640 or complete CDM, next day collected by centrifugation, washed 4 x with DPBS and reinoculated in the appropriate media (RPMI1640 or complete CDM, respectively; or complete CDM overnight cultures for SNM samples). Samples were harvested after 24 or 48 hours (for all CDMs lacking Val). For experiments conducted in TSB medium (Fig 1), aureusimines were purified from 24-hour cultures. OD_{600}=6 equivalents were collected for all samples. Bacterial supernatants were collected by centrifugation at 6,200 x g for 10 min at 4°C and sterilized by filtration through a 0.20 µm pore-size filter. 2-10 mL of filtrate were mixed in glass vials with equal parts of 100% HPLC-grade chloroform (Carl Roth, Cat. No. 7331.2) and mixed by vortexing. Samples were centrifuged at 3, 000 x g for 10 min at 4°C and the upper, organic phase was carefully transferred to 1.5 mL HPLC-grade glass vials. Vehicle was evaporated in an extractor hood using a continuous flow of pressurized air. Dried samples were solubilized in 100 µL 20% (v/v) DMSO. Ultra-performance liquid chromatography coupled with mass spectrometry (UPLC-MS) measurements were carried out as described [7]. Commercially available phevalin/aureusimine B [CAS Number 170713-71-0] (Biozol, Cat. No. TRC-A794570) and tyrvalin/aureusimine A [CAS Number 1244033-70-2] (Biozol, Cat. No. TRC-A794560) were used as reference.

Metabolomic analysis

All experiments were performed in biological triplicates (n=3) and technical duplicates. Bacteria were grown in the appropriate culture medium (RPMI1640 or CDM) as indicated (24 or 48h). Supernatants and pellets were collected separately, heat inactivated for 20 min at 95°C, followed by freezing at -80°C. For metabolite measurements, all chemicals were purchased from Sigma Aldrich at the highest purity available.

For **supernatant** measurements, 100 µl of the supernatants were spiked with 10 nmol norvaline as internal standard and were dried under a gentle stream of nitrogen. For derivatization, 50 µl water-free acetonitrile and 50 µl of MTBSTFA (N-(tert-butyldimethyl-silyl)-N-methyl-trifluoroacetamide containing 1% tert-butyl-dimethyl-silylchlorid) were added and the sample was heated at 70°C for 45 min. The tert-butyldimethylsilyl (TBDMS) derivatives were then analyzed by gas chromatography-mass spectrometry (GC-MS). Analysis was performed with a QP2010 Plus gas chromatograph/mass spectrometer (Shimadzu) equipped with a fused silica capillary column (Equity TM-5; 30 m × 0.25 mm, 0.25µm film thickness; SUPELCO) and a quadrupole detector working with electron impact ionization

at 70 eV. An aliquot of the derivatized samples was injected in 1:5 split mode at an interface temperature of 260°C and a helium inlet pressure of 70 kPa. After sample injection, the column was first kept at 150°C for 3 min and then developed with a temperature gradient of 7°C min^{-1} to a final temperature of 300°C. This temperature was held for further 3 min. Each sample was measured twice. Valine (R_t 9.1 min), phenylalanine (R_t 15.5 min) and tyrosine (22.1 min) were calculated relative to norvaline (R_t 9.3 min).

For measurement of **bacterial pellets,** freeze dried bacterial cell pellets (approx. $6x10^6$ cells) were suspended in 1 ml methanol and mechanically disrupted using a ribolyser (3 × 20 s; 6.5 m s−1). The solution was then centrifuged at 10,000 x g for 20 min at 4 °C. This procedure was performed twice. The supernatants were combined, spiked with 10 nmol norvaline as internal standard and then dried under N_2 flux. The residue was treated with 50 µl MTBSTFA and 50 µl water-free acetonitrile at 70 °C for 45 min. The tert-butyldimethylsilyl (TBDMS) derivatives were then analyzed by GC-MS (conditions as above).

Statistical analysis

Use of statistical analysis is indicated in the corresponding figure legend. No matching or pairing was assumed in the experimental design. Gaussian distribution of residuals and equal variance were tested by Shapiro-Wilk and Brown-Forsythe test, respectively, prior to running a parametric test.

Supplemental references

1. Schoenfelder, S.M.K., et al., *Methionine Biosynthesis in Staphylococcus aureus Is Tightly Controlled by a Hierarchical Network Involving an Initiator tRNA-Specific T-box Riboswitch.* PLOS Pathogens, 2013. **9**(9): p. e1003606.
2. Krismer, B., et al., *Nutrient Limitation Governs Staphylococcus aureus Metabolism and Niche Adaptation in the Human Nose.* PLOS Pathogens, 2014. **10**(1): p. e1003862.
3. Fey, P.D., et al., *A Genetic Resource for Rapid and Comprehensive Phenotype Screening of Nonessential Staphylococcus aureus Genes.* mBio, 2013. **4**(1): p. e00537-12.
4. Kreiswirth, B.N., et al., *The toxic shock syndrome exotoxin structural gene is not detectably transmitted by a prophage.* Nature, 1983. **305**(5936): p. 709-712.
5. Schoenfelder, S.M.K., et al., *Antibiotic resistance profiles of coagulase-negative staphylococci in livestock environments.* Veterinary Microbiology, 2017. **200**: p. 79-87.
6. Bae, T. and O. Schneewind, *Allelic replacement in Staphylococcus aureus with inducible counter-selection.* Plasmid, 2006. **55**(1): p. 58-63.
7. Blättner, S., et al., *Staphylococcus aureus Exploits a Non-ribosomal Cyclic Dipeptide to Modulate Survival within Epithelial Cells and Phagocytes.* PLOS Pathogens, 2016. **12**(9): p. e1005857.
